# P05.32. A tool for rapid identification of potential herbal medicine-drug interactions 2011 update: a review

**DOI:** 10.1186/1472-6882-12-S1-P392

**Published:** 2012-06-12

**Authors:** L Girard, A Kutt, C Necyk, S Jasser, B Law, A Filipelli, P Gardiner, H Boon, S Vohra

**Affiliations:** 1 CARE Program, Edmonton, Alberta, Canada; 2 School Of Public Health, Boston University, Boston, USA; 3 Department of Family Medicine, Boston University School of Medicine, Boston, USA; 4 Leslie Dan Faculty of Pharmacy, University of Toronto, Toronto, Canada

## Purpose

Individuals using herbal medicines concomitantly with prescription drugs are at risk of harms due to herb-drug interactions. As part of a prospective active surveillance study to identify natural health product harms, a tool to identify herb-drug interactions was created for clinician use. Such tools require regular updating to remain clinically relevant. Our objective was to review the herb-drug literature and update the herb-drug interaction grid.

## Methods

Herbs and drugs reviewed were based on the prevalence of their use. Database searches for herbs were conducted in MEDLINE, EMBASE, and IPA between 2007 and 2010. Herbs were searched with the following terms: ‘clinical trials’, ‘case studies’, and ‘case reports’. Abstracts of each article were read to identify herb-drug interactions. All potential interactions were reviewed by an expert (PG or HB). Reference lists of relevant review articles were analyzed for additional papers, as was the textbook Herb, Nutrient, and Drug Interactions: clinical implications and therapeutic strategies. Data extraction involved classifying the interactions into four groups: (1) No reported or theoretical interactions, (2) Theoretical interactions based on animal or in vitro data, (3) Theoretical interactions extrapolated from clinical data, and (4) Interactions supported by clinical evidence.

## Results

Two thousand one hundred forty-eight references were identified by the searches, and 117 potential updates are being sent to reviewers.

## Conclusion

The herbal medicine-drug interaction grid will allow clinicians to have a guide on potential herbal medicine-drug harms based on the most recent literature.

